# Copper oxide nanoparticles: Synthesis, toxic potential and modulation of astrocytic metabolism

**DOI:** 10.1186/2193-1801-4-S1-P5

**Published:** 2015-06-12

**Authors:** Felix Bulcke, Ralf Dringen, Karsten Thiel

**Affiliations:** Centre for Biomolecular Interactions Bremen (CBIB), University of Bremen, Germany; Fraunhofer Institute for Manufacturing Technology and Advanced Materials, Germany

**Keywords:** Copper, Nanoparticles, Astrocytes

To test for potential consequences of an exposure of brain cells to copper oxide nanoparticles (CuO-NPs), we have synthesized dimercaptosuccinate-coated CuO-NPs. These particles had a diameter of around 5 nm as determined by transmission electron microscopy but were dispersed as aggregate as demonstrated by their average hydrodynamic diameter in aqueous dispersion of 136 ± 4 nm. Exposure of cultured primary astrocytes to CuO-NPs increased the cellular copper levels and compromised the cell viability in a time- and concentration-dependent manner. CuO-NPs in concentrations above 100 µM (6.3 µg copper/mL) severely affected the viability of the cells, as demonstrated by a lowered tetrazolium dye reduction capacity, a lowered cellular lactate dehydrogenase activity, a increased membrane permeability and the generation of reactive oxygen species. In contrast, exposure of astrocytes for 24 h with 100 µM CuO-NPs did hardly affect the viability of astrocytes but stimulated the glycolytic flux, increased the cellular glutathione content, stimulated the release of glutathione and elevated the level of the metal storing proteins metallothioneins. Presence of the intracellular copper chelator tetrathiomolybdate throughout the incubation with CuO-NPs protected the cells against the toxicity of CuO-NPs and prevented the stimulation of the glycolytic flux as well as the increased levels of metallothioneins. These data demonstrate that CuO-NPs can severely damage cultured astrocyes and that copper ions derived from sub-toxic concentrations of CuO-NPs strongly affected the metabolism of astrocytes.

